# Urinary Concentrations of Metabolites of Pyrethroid Insecticides in the General U.S. Population: National Health and Nutrition Examination Survey 1999–2002

**DOI:** 10.1289/ehp.0901275

**Published:** 2010-02-03

**Authors:** Dana Boyd Barr, Anders O. Olsson, Lee-Yang Wong, Simeon Udunka, Samuel E. Baker, Ralph D. Whitehead, Melina S. Magsumbol, Bryan L. Williams, Larry L. Needham

**Affiliations:** National Center for Environmental Health, Centers for Disease Control and Prevention, Atlanta, Georgia, USA

**Keywords:** general population, insecticide, NHANES, pyrethroid, 3-phenoxybenzoic acid, urine

## Abstract

**Background:**

Pyrethroid insecticides are the most commonly used residential insecticides in the United States.

**Objectives:**

Our objective was to assess human exposure via biomonitoring to pyrethroid insecticides in a representative sample of the general U.S. population ≥ 6 years of age.

**Methods:**

By using isotope-dilution high-performance liquid chromatography/electrospray chemical ionization/tandem mass spectrometry, we measured five urinary metabolites of pyrethroid insecticides in 5,046 samples collected as a part of the 1999–2002 National Health and Nutrition Examination Survey (NHANES). Univariate, multivariate, and Pearson correlation analyses were performed using SUDAAN and SAS software, incorporating the appropriate sample weights into the analyses. Multivariate analyses included age, sex, race/ethnicity, creatinine, fasting status, and urine collection time as covariates.

**Results:**

We detected 3-phenoxybenzoic acid (3PBA), a metabolite common to many pyrethroid insecticides, in more than 70% of the samples. The least-squares geometric mean (LSGM) concentration (corrected for covariates) of 3PBA and the frequency of detection increased from 1999–2000 (0.292 ng/mL) to 2001–2002 (0.318 ng/mL) but not significantly. Non-Hispanic blacks had significantly higher LSGM 3PBA concentrations than did non-Hispanic whites and Mexican Americans in the 2001–2002 survey period and in the combined 4-year survey periods but not in the 1999–2000 survey period. Children had significantly higher LSGM concentrations of 3PBA than did adolescents in both NHANES periods and than adults in NHANES 1999–2000. *Cis*- and *trans*-(2,2-dichlorovinyl)-2,2-dimethylcyclopropane-1-carboxylic acid were highly correlated with each other and with 3PBA, suggesting that urinary 3PBA was derived primarily from exposure to permethrin, cypermethrin, or their degradates.

**Conclusions:**

Pyrethroid insecticide exposure in the U.S. population is widespread, and the presence of its metabolites in the urine of U.S. residents indicates that children may have higher exposures than adolescents and adults.

Synthetic pyrethroid insecticides are a major class of insecticides that are replacing home and some agricultural use of organophosphorus and carbamate insecticides [Agency for Toxic Substances and Disease Registry (ATSDR) 2003]. They are human-made derivatives of pyrethrins, which are naturally occurring insecticides found in a variety of plants such as chrysanthemums (ATSDR 2003). Synthetic pyrethroids are designed to be more chemically potent and environmentally stable than natural pyrethrins while still retaining their relatively low mammalian toxicity (Soderlund et al. 2002). Although they were not used commercially until 1980 (Aprea et al. 1997), by 1982 pyrethroid insecticides accounted for more than 30% of the world market (Aprea et al. 1997; Leng et al. 1997a). Pyrethroid insecticides are the most widely used agents for indoor pest control (Go et al. 1999). Those developed since the early 1970s have improved photostability and minimal volatility, which have enabled their successful use in widespread agricultural applications (Laws and Hayes 1991). Although > 1,000 pyrethroids have been designed (ATSDR 2003), the U.S. Environmental Protection Agency (EPA) has approved use of only about 20 pyrethroid insecticides (U.S. EPA 1991), and less than a dozen are used with any frequency in the United States. Permethrin is the most commonly used pyrethroid insecticide in the United States (ATSDR 2003).

Like many other classes of insecticides, the pyrethroid insecticides are acute neurotoxicants (Aldridge 1990; Bradbury and Coats 1989; Vijverberg and van den Bercken 1990), and although dissimilar in structure and environmental and biological persistence to *p*,*p*′-dichlorodiphenyltrichloroethane (DDT), their modes of action are strikingly similar (Vijverberg and van den Bercken 1990; Vijverberg et al. 1982). Like DDT, pyrethroid insecticides modulate the function of voltage-gated sodium channels (Vijverberg et al. 1982); however, pyrethroid insecticides, unlike DDT, have short biological half-lives, in the order of hours, whereas DDT has a half-life of many years. Specifically, pyrethroid insecticides alter the permeability of excited nerve cells to sodium ions and cause repetitive nerve impulses that can vary between a few dozen for the less toxic non-cyano-substituted pyrethroid insecticides (type I) to up to 1,000 for the more toxic cyano- substituted pyrethroid insecticide (type II) (Aldridge 1990; Bradbury and Coats 1989; Vijverberg and van den Bercken 1990). They also have other neurobiologic actions, including effects on central γ-amino butyric acid, noradrenergic, dopaminergic, and cholinergic neurotransmission (Mandhane and Chopde 1997). In general, pyrethroid insecticides are considered among the lower-human-toxicity insecticides because mammals have higher levels than do insects of the enzymes that detoxify pyrethroid insecticides; thus, pyrethroid insecticides are rapidly metabolized and excreted in mammalian systems (Narahashi 2001).

Although studies in humans demonstrate that pyrethroid insecticides are absorbed readily after exposure by inhalation, oral, and dermal routes (ATSDR 2003), the primary exposure route in the general population is thought to be through dietary intake (ATSDR 2003). However, because of their burgeoning use in common household insecticide products such as spray insecticides, aerosol bombs, and pet shampoos (ATSDR 2003), low-level episodic exposures from household use are probable. Exposures from household use in colder areas likely occur in the spring and summer when household pests are at their peak; however, in warmer climates, pyrethroid insecticides may be used in homes year-round. Furthermore, the use of pyrethroid insecticides, particularly permethrin, in lice treatments and shampoos may allow for direct exposure to certain pyrethroid insecticides in children.

Mammalian animal studies have identified several important metabolites of pyrethroid insecticides. 3-Phenoxybenzoic acid (3PBA) is a metabolite of many pyrethroid insecticides resulting from the oxidation of the hydrolytic product of many pyrethroid insecticides, 3-phenoxybenzyl alcohol (Figure 1, Table 1). Similarly, 4-fluoro-3-phenoxybenzoic acid (4F3PBA) is a metabolite of the fluorine-substituted pyrethroid insecticide cyfluthrin. Chrysanthemic acid derivatives are also obtained after pyrethroid insecticide metabolism (Figure 1). *Cis*- and *trans*-(2,2-dichlorovinyl)-2,2-dimethylcyclopropane-1-carboxylic acid (*cis*- and *trans*-DCCA) are metabolites of the chlorinated pyrethroid insecticides permethrin, cypermethrin, and cyfluthrin. *Cis*-(2,2-dibromovinyl)-2,2-dimethylcyclopropane-1-carboxylic acid (*cis*-DBCA) is a selective metabolite of deltamethrin. Human dosing studies of cypermethrin isomers (Eadsforth and Baldwin 1983; Eadsforth et al. 1988; Woollen et al. 1992) and cyfluthrin (Leng et al. 1997b) and occupational exposure studies (Aprea et al. 1997; Hardt and Angerer 2003; Leng et al. 1996) have confirmed that many of these metabolites are important markers of pyrethroid insecticide exposure in humans (Leng et al. 1997b). The presence of metabolites representing both halves of the pyrethroid insecticide molecule can indicate the source pyrethroid insecticides for the metabolites.

We report urinary concentrations of five metabolites of pyrethroid insecticides in 1,998 persons of the general U.S. population 6–59 years of age in 1999 and 2000 and in 3,048 persons ≥ 6 years of age in 2001 and 2002. Specifically, we report urinary concentrations of 3PBA, a common metabolite of up to 18 synthetic pyrethroid insecticides; 4F3PBA, a metabolite of cyfluthrin; *cis*-DCCA and *trans*-DCCA, geometric isomeric metabolites of permethrin, cypermethrin, and cyfluthrin; and *cis*-DBCA, a metabolite of deltamethrin (Figure 2). The data we report are representative of the civilian, noninstitutionalized U.S. population and are stratified by age, sex, and race/ethnicity. We also evaluated fasting duration and time of sample collection (i.e., morning, afternoon, evening) to see if the metabolite concentrations may have been influenced by these factors.

## Methods

### Study population

The National Health and Nutrition Examination Survey (NHANES), conducted by the National Center for Health Statistics (NCHS) of the Centers for Disease Control and Prevention (CDC), is designed to measure the health and nutrition status of the civilian noninstitutionalized U.S. population (CDC 2003). In 1999, NHANES became a continuous survey, fielded on an ongoing basis. Each year of data collection is based on a representative sample, which covers all ages of the civilian noninstitutionalized population. Public-use data files have been released in 2-year groupings (cycles). In this study, national population estimates for pyrethroid metabolites and estimates for the three largest race/ethnicity subgroups in the U.S. population (non-Hispanic white, non-Hispanic black, and Mexican American) are derived from two 2-year cycles of the survey, NHANES 1999–2000 and NHANES 2001–2002. The study was reviewed and approved by the Ethical Review Board at the NCHS and complied with all national and international guidelines on research involving human subjects.

The sampling scheme for NHANES is based on a complex multistage area probability design, which includes selection of primary sampling units (counties), household segments within the counties, and sample persons from selected households. In 1999 and 2000, people 12–19 years and ≥ 60 years of age, non-Hispanic blacks, and Mexican Americans were oversampled. Low-income white Americans were oversampled beginning in 2000. Data were collected through a household interview and a standardized physical examination that was conducted in a mobile examination center. Spot urine specimens were collected from each participant ≥ 6 years of age during one of three daily examination periods. Sociodemographic information and medical histories of the survey participant and the family were collected during the household interview. Fasting duration was also collected from each participant.

NHANES 1999–2000 was conducted in 26 locations throughout the United States and included examinations of 9,282 people; NHANES 2001–2002 was conducted in 30 locations and included examinations of 10,000 people. For the pyrethroid metabolites in the first NHANES cycle, we conducted measurements on a subset of participants based on a random one-half sample of children 6–11 years of age in 1999 and 2000, a random one-quarter sample of people 12–59 years of age in 1999, and a random one-third sample of people 12–59 years of age in 2000. For the second NHANES cycle, we conducted measurements on a subset of participants based on a one-half sample of children 6–11 years of age in 2001, a random one-third sample of children 6–11 years of age in 2002, and a random one-third sample of people ≥ 12 years of age in 2001 and 2002. Because the subset was a random selection from the entire set, the ability of the samples tested to accurately represent the U.S. population was maintained.

### Laboratory methods

The urine for pyrethroid measurements was aliquoted at the collection site, stored cold (2–4°C) or frozen until shipment to CDC’s National Center for Environmental Health laboratory on dry ice. Urinary creatinine concentrations were determined using an automated colorimetric method based on a modified Jaffe reaction (Jaffe 1886) on a Beckman Synchron AS/ASTRA clinical analyzer (Beckman Instruments, Inc., Brea, CA) at the Fairview University Medical Center (Minneapolis, MN). We analyzed urine samples for pyrethroid metabolites using established methodology (Baker et al. 2004; Olsson et al. 2004). Briefly, 2 mL urine was spiked with an internal standard mixture consisting of isotopically labeled 3PBA and *trans-*DCCA and incubated with β-glucuronidase/sulfatase to liberate conjugated metabolites. The hydrolysates were extracted using OASIS HLB (Waters Corp., Milford, MA) mixed-mode solid-phase extraction cartridges. The cartridges were washed with 5% methanol in a 0.1% acetic acid solution, and the metabolites were eluted using methanol. The extracts were concentrated and analyzed using high-performance liquid chromatography/electrospray chemical ionization/tandem mass spectrometry. 3PBA and *trans-*DCCA were quantified using isotope dilution calibration, whereas 4F3PBA, *cis-*DCCA, and *cis*-DBCA were quantified using the labeled 3PBA, labeled *trans*-DCCA, and labeled *trans*-DCCA, respectively, as internal standards. Positive and negative control samples represented 10% of the samples analyzed to ensure proper method operation. Metabolite concentrations were adjusted using creatinine concentrations to correct for variable urine dilutions in the “spot” urine samples. Both laboratories and methods were certified according to guidelines set forth in the Clinical Laboratory Improvement Amendment of 1988.

### Demographic and other covariates

Age was reported at the time of the household interview as the age in years at the last birthday. Age categories used in our statistical analyses were 6–11, 12–19, and 20–59 years for 1999–2002, and ≥ 60 years for 2001–2002. A composite race/ethnicity variable based on self-reported race and ethnicity was created to define three major racial/ethnic groups: non-Hispanic black, non-Hispanic white, and Mexican American. Individuals from other racial/ethnic groups were included in the total estimates reported here; however, no separate demographic breakdown is provided.

Traditionally, creatinine concentrations have been used to adjust spot urine samples for variable dilution caused by the different hydration states of the sample donor. Because creatinine concentrations vary with age, sex, and race/ethnicity, creatinine adjustment in diverse populations would not be valid for comparisons of pyrethroid insecticide metabolite concentrations among the demographic groups (Barr et al. 2005). To overcome this limitation and allow for an appropriate comparison of metabolite concentrations among the demographic groups, we also used creatinine as a covariate in statistical models (Barr et al. 2005). By using this model, we appropriately corrected for covariate effects on the creatinine concentrations while reducing the variability caused by urine dilution of spot samples.

### Statistical analysis

Survey-specific sample weights calculated for the specified random subset were used in statistical analyses. SUDAAN (RTI International, Research Triangle Park, NC) incorporates the NHANES sampling weights and adjusts for the complex sample design of the survey. Sample weights take into account the unequal probabilities of selection, resulting from the cluster design and the planned oversampling of certain subgroups. Oversampling of adolescents, the elderly, non-Hispanic blacks, and Mexican Americans necessitated the use of sampling weights in all analyses to produce national estimates of prevalence and associated variances.

Parametric statistics were performed only on metabolites with sufficient frequency of detection (> 60%) to avoid undue influence on the estimates caused by imputed values in the analyses; thus, 3PBA was the only metabolite parametrically analyzed. Data distribution, univariate, bivariate, and multivariate statistics were evaluated. Arithmetic means, geometric means (GMs), least-squares geometric means (LSGMs), weighted Pearson correlation coefficients, and percentiles of 3PBA, *cis*-DCCA, and *trans*-DCCA concentrations were calculated using SAS (release 9.1; SAS Institute Inc., Cary, NC) and SUDAAN (release 9.0). For comparing populations, LSGMs corrected the GMs for the common statistical variables tested (i.e., age group, race/ethnicity, sex, and time of sample collection, and fasting) that showed correlations in bivariate statistics or could potentially influence comparisons. For concentrations below the analytic limits of detection (LODs), a value equal to the LOD divided by the square root of 2 was used (Hornung and Reed 1990). The LODs were 0.1 ng/mL for 3PBA, 0.2 ng/mL for 4F3PBA, 0.1 ng/mL for *cis*-DCCA, 0.4 ng/mL for *trans*-DCCA, and 0.1 ng/mL for *cis*-DBCA. Differences in LSGMs among demographic groups were considered to be significant when *p* < 0.05.

## Results

Table 2 shows distribution percentiles of concentrations of 3PBA, both volume based and creatinine adjusted. Of the metabolites measured, 3PBA was the most frequently detected. Overall, 3PBA was detected more frequently in samples collected in 2001 and 2002 (75.4%) than in samples collected in 1999–2000 (66.5%; *p* = 0.07). Univariate analysis results showed that the GM of the 3PBA concentrations among race (*p* = 0.003 for 1999–2000; *p* < 0.001 for 2001–2002) and age group (*p* = 0.003 for 1999–2000 only) were significantly different.

**Figure 3:**
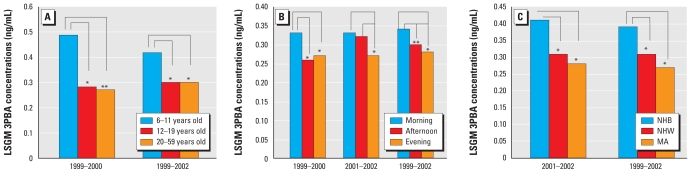
LSGM concentrations of age (*A*), sample collection time (*B*), and race (*C*: MA, Mexican American; NHB, non-Hispanic black; NHW, non-Hispanic white). *p < 0.05. **0.05 < *p* < 0.1.

For the 1999–2000 survey period, in the final multivariate model, age group and time of sample collection were the only significant variables in addition to log-transformed creatinine. The LSGM 3PBA concentration was higher for children than for adolescents (*p* = 0.009) (Figure 3A). Samples collected during the morning sampling period had significantly higher 3PBA concentrations than did those collected in the afternoon (*p* = –0.002) and evening (*p* < 0.001) sampling periods (Figure 3B).

For the 2001–2002 survey period, race (*p* = 0.008; Figure 3C) and sample collection time (*p* = 0.036; Figure 3B) were significant in the final model, but not age group or sex. The LSGM 3PBA concentration of non-Hispanic blacks was higher than those of non-Hispanic whites (*p* = 0.039) and Mexican Americans (*p* = 0.025) (Figure 3C). 3PBA concentrations in samples collected during the evening sampling period were significantly lower than those collected in the morning (*p* = 0.001) and afternoon (*p* = 0.007) (Figure 3B).

For combined survey periods (1999–2002), race, age, and time of collection were all significant in the final model. Non-Hispanic blacks had a higher LSGM 3PBA concentrations than did non-Hispanic whites (*p* = 0.0096) and Mexican Americans (*p* < 0.0001). Children had higher LSGM 3PBA concentration than did adolescents (*p* = 0.001) and adults (*p* = 0.0014); however, we observed no difference between adolescents and adults. 3PBA concentrations in samples collected during the evening sampling period were significantly lower than those collected in the morning (*p* = 0.02) and afternoon (*p* = 0.03; Figure 3B). Fasting duration was not a significant variable in either survey period or in the combined survey periods.

Tables 3 and 4 show distribution percentiles of concentrations of *cis*- and *trans*-DCCA, which were highly correlated with each other (*r* = 0.887; *p* < 0.001) and with 3PBA (*r* = 0.766; *p* = 0.02). However, the ratio of the *cis*- and *trans*-isomers of DCCA varied.

4F3PBA was detected in 3.2% and 0.6% of the urine samples analyzed in 1999–2000 and 2001–2002, respectively. Similarly, *cis-*DBCA was detected in 1.3% and 0.5% of the urine samples analyzed in 1999–2000 and 2001–2002, respectively. Both 4F3PBA and *cis-*DBCA were detected in too few of the samples tested to allow a reliable estimation of their distribution percentiles and GM concentrations.

## Discussion

Although pyrethroid insecticides have largely replaced organophosphorus and carbamate insecticides as the most commonly used household insecticides, these are the first population-based data reported for the general U.S. population. Because these pesticides are relatively nonvolatile, the primary source of exposure is believed to be through diet, with perhaps short episodes of exposure from residential use of insect control products. Children had higher concentrations of pyrethroid insecticide metabolites than did adolescents and adults. These results are consistent with many other pesticide-related exposures and probably reflect differences in diet and behaviors (e.g., hand-to-mouth behaviors). Non-Hispanic blacks had higher concentrations than did the other race/ethnicity groups. This observation could potentially be an indicator of socioeconomic or housing status. Older homes and lower income households are likely to have more pests, requiring use of more household pesticides. Dietary differences could also account for the higher levels of pyrethroid pesticide metabolites.

Concentrations of pyrethroid metabolites tended to be higher during the morning collection periods than during the afternoon or evening collection periods. We cannot plausibly explain this observation; however, this may underscore the need to have consistent collection times in future studies.

The high correlation between 3PBA and *cis*- and *trans*-DCCA suggests that the parent pesticides resulting in U.S. population exposures were predominantly permethrin or cypermethrin, which is consistent with U.S. pesticide use data. The low frequency of detection of 4F3PBA and *cis*-DBCA suggests that exposure to cyfluthrin and deltamethrin is infrequent.

Interestingly, the ratio of *trans*-DCCA and *cis*-DCCA varied from 0.001 to > 5,800; however, the vast majority of our ratios ranged between 3 and 4. The ratio of *trans*- to *cis*-DCCA could vary based upon a variety of criteria, including product formulation, differential metabolism, different half-lives in environmental media, different parent chemicals from which it was derived, exposure routes, and other factors.

The only population-based data on these metabolites were generated in Germany during the last decade (Becker et al. 2006; Heudorf and Angerer 2001; Heudorf et al. 2006). Generally, the U.S. population-based data are consistent with those from the German studies, where median values of 3PBA ranged from 0.04 ng/mL to 0.29 ng/mL, compared with our median levels of 0.25 ng/mL and 0.27 ng/mL for NHANES 1999–2000 and 2001–2002, respectively. The frequencies of detection were relatively similar for *cis*- and *trans*-DCCA concentrations in the German studies and in our study of the U.S. population. 4F3PBA and *cis*-DBCA were detected more frequently in the German studies than in our U.S. study but were detected much less frequently than the other metabolites, similar to our data.

Interestingly, Schettgen et al. (2002) evaluated the population-based levels of 3PBA and the DCCA isomers along with other exposure factors and concluded that the German population-based concentrations were likely from dietary exposure. We can speculate a similar source for our U.S. population-based data; however, supplementary dietary exposure data must be obtained to verify this supposition. Because residential exposures are likely periodic and vary over time, our large population sample probably minimized the contribution of these “spikes” in exposure, leaving baseline exposure concentrations, likely from the continual dietary contributions to pyrethroid exposures.

## Conclusions

We report the first population-based biomonitoring data for pyrethroid metabolites for the U.S. population for the period 1999–2002. Pyrethroid pesticide exposure appears widespread, likely because of a shift in home pesticide use from the organophosphorus insecticides. Our data show that non-Hispanic blacks have significantly higher concentrations than do non-Hispanic whites and Mexican Americans of a common pyrethroid metabolite (3PBA) in the 2001–2002 survey period and in the combined 4-year survey period but not in the 1999–2000 survey period. Children had significantly higher concentrations of 3PBA than did adolescents in both NHANES survey periods and than did adults in NHANES 1999–2000. These data will be useful to establish reference range concentrations for these metabolites and for evaluating changes in pyrethroid exposure over time in the United States. The U.S. population-based levels we found are comparable to the levels found in studies conducted in Germany.

## Figures and Tables

**Figure 1:**
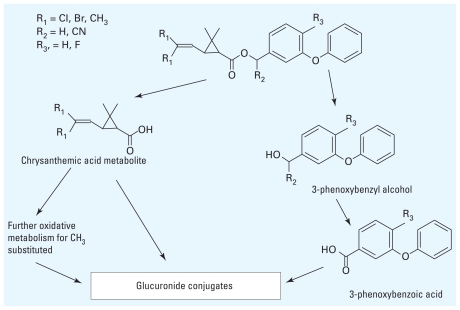
General metabolism of both type I and type II pyrethroid insecticides. Type I insecticides have an R_2_ substitution of H, and type II insecticides have an R_2_ substitution of CN (cyano group).

**Figure 2:**
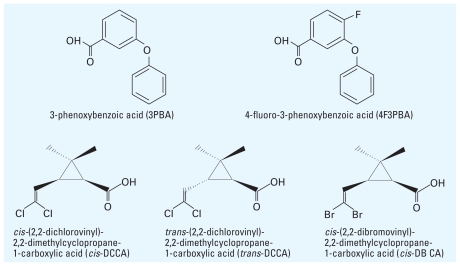
Pyrethroid metabolites measured in NHANES 1999–2002.

**Table 1:** Common pyrethroid insecticides and their corresponding urinary metabolites.

	Metabolite				
Pyrethroid insecticide	3PBA	*cis*-DCCA	*trans*-DCCA	4F3PBA	*cis*-DBCA
Permethrin	X	X	X		
Cypermethrin	X	X	X		
Cyfluthrin		X	X	X	
Deltamethrin	X				X
Allethrin	X				
Resmethrin	X				
Fenvalerate	X				

Some metabolites are excreted as glucuronide conjugates, which we liberated before analysis; only the liberated metabolites are listed here.

**Table 2:** Urinary concentrations of 3PBA in the general U.S. population, NHANES 1999–2002.

Category/survey years	GM (95% confidence interval)	Selected percentile (95% confidence interval)				Sample size (*n*)	Weighted detection (%)
		50th	75th	90th	95th		
Total							
1999–2000^a^	0.292 (0.247–0.345)	0.25 (0.19–0.32)	0.73 (0.59–0.85)	1.75 (1.49–2.16)	4.33 (2.62–6.30)	1,998	66.5
2001–2002^a^	0.318 (0.275–0.368)	0.27 (0.22–0.34)	0.70 (0.57–0.81)	1.73 (1.49–2.16)	3.54 (2.69–5.25)	3,048	75.4
1999–2000^b^	0.261 (0.224–0.304)	0.25 (0.20–0.28)	0.55 (0.46–0.63)	1.40 (1.13–1.73)	3.19 (2.16–4.55)	1,998	66.5
2001–2002^b^	0.324 (0.284–0.371)	0.29 (0.25–0.34)	0.60 (0.51–0.75)	1.54 (1.26–1.91)	3.35 (2.50–4.92)	3,046	75.4

Age group							
6–11 years							
1999–2000^a^	0.417 (0.292–0.595)	0.32 (0.21–0.49)	10.12 (0.70–1.60)	4.18 (2.02–6.54)	8.63 (3.89–71.1)	483	71.9
2001–2002^a^	0.325 (0.260–0.406)	0.30 (0.20–0.42)	0.76 (0.57–1.05)	1.81 (1.42–2.78)	3.38 (2.25–4.12)	580	75.2
1999–2000^b^	0.450 (0.299–0.677)	0.30 (0.19–0.44)	0.89 (0.57–1.35)	2.59 (1.41–5.49)	7.20 (1.88–64.0)	483	71.9
2001–2002^b^	0.423 (0.335–0.534)	0.33 (0.25–0.41)	0.66 (0.49–1.06)	1.81 (1.28–2.58)	3.04 (2.00–4.09)	580	75.2

12–19 years							
1999–2000^a^	0.336 (0.265–0.427)	0.29 (0.20–0.44)	0.87 (0.62–1.04)	1.93 (1.49–2.90)	4.33 (1.83–11.1)	682	73.2
2001–2002^a^	0.353 (0.288–0.434)	0.30 (0.25–0.39)	0.80 (0.56–1.13)	1.86 (1.48–2.35)	3.45 (2.14–6.69)	831	79.8
1999–2000^b^	0.227 (0.178–0.290)	0.16 (0.12–0.20)	0.39 (0.29–0.52)	1.16 (0.71–1.39)	1.65 (1.25–4.21)	682	73.2
2001–2002^b^	0.274 (0.229–0.328)	0.21 (0.18–0.25)	0.46 (0.35–0.61)	1.01 (0.72–1.46)	1.69 (1.10–5.48)	830	79.8

20–59 years							
1999–2000^a^	0.267 (0.227–0.314)	0.23 (0.16–0.30)	0.64 (0.51–0.82)	1.49 (1.25–1.78)	3.21 (2.04–5.41)	833	64.2
2001–2002^a^	0.314 (0.271–0.364)	0.27 (0.22–0.34)	0.67 (0.53–0.78)	1.65 (1.27–2.34)	3.25 (2.51–6.16)	1,128	75.8
1999–2000^b^	0.246 (0.216–0.278)	0.26 (0.22–0.29)	0.55 (0.46–0.63)	1.30 (1.04–1.62)	2.86 (1.97–4.18)	833	64.2
2001–2002^b^	0.311 (0.271–0.357)	0.30 (0.26–0.35)	0.60 (0.50–0.75)	1.59 (1.15–2.46)	3.43 (2.37–5.11)	1,128	75.8

≥ 60 years							
2001–2002^a^	0.303 (0.233–0.394)	0.25 (0.18–0.35)	0.69 (0.49–1.10)	2.03 (1.45–3.68)	5.16 (2.77–6.61)	509	70.3
2001–2002^a^	0.372 (0.301–0.462)	0.32 (0.24–0.41)	0.73 (0.54–1.02)	2.16 (1.28–3.50)	4.06 (2.44–6.15)	508	70.3

Sex							
Male							
1999–2000^a^	0.273 (0.226–0.330)	0.25 (0.18–0.33)	0.71 (0.57–0.82)	1.49 (1.29–1.73)	2.41 (1.92–3.79)	974	65.3
2001–2002^a^	0.326 (0.281–0.379)	0.30 (0.23–0.36)	0.69 (0.57–0.75)	1.60 (1.37–2.15)	3.23 (2.56–5.25)	1,429	77.8
1999–2000^b^	0.210 (0.173–0.253)	0.19 (0.15–0.25)	0.51 (0.39–0.59)	1.13 (0.86–1.49)	1.85 (1.49–2.53)	974	65.3
2001–2002^b^	0.269 (0.233–0.310)	0.24 (0.20–0.28)	0.50 (0.43–0.58)	1.28 (1.05–1.59)	3.00 (1.74–4.00)	1,429	77.8

Female							
1999–2000^a^	0.311 (0.253–0.384)	0.25 (0.19–0.34)	0.74 (0.51–0.99)	2.30 (1.63–3.36)	6.03 (3.27–11.8)	1,024	67.7
2001–2002^a^	0.311 (0.260–0.371)	0.25 (0.20–0.32)	0.73 (0.55–0.92)	1.81 (1.53–2.47)	3.76 (2.51–6.55)	1,619	73.1
1999–2000^b^	0.323 (0.270–0.387)	0.28 (0.24–0.34)	0.60 (0.47–0.71)	1.88 (1.31–2.86)	5.03 (3.08–6.90)	1,024	67.7
2001–2002^b^	0.388 (0.331–0.455)	0.33 (0.28–0.41)	0.73 (0.56–0.93)	1.84 (1.45–2.35)	4.43 (2.62–5.59)	1,617	73.1

Race/ethnicity							
Non-Hispanic white							
1999–2000^a^	0.288 (0.233–0.355)	0.24 (0.16–0.32)	0.71 (0.53–0.85)	1.78 (1.41–3.05)	5.34 (2.62–8.43)	603	65.1
2001–2002^a^	0.297 (0.248–0.355)	0.24 (0.19–0.32)	0.60 (0.49–0.78)	1.75 (1.46–2.34)	3.70 (2.52–6.16)	1,281	73
1999–2000^b^	0.272 (0.225–0.329)	0.28 (0.23–0.34)	0.63 (0.51–0.80)	1.88 (1.35–2.53)	4.44 (2.29–5.93)	603	65.1
2001–2002^b^	0.321 (0.274–0.377)	0.31 (0.26–0.36)	0.67 (0.53–0.91)	1.84 (1.46–2.62)	4.43 (2.63–6.04)	1,280	73

Mexican American							
1999–2000^a^	0.260 (0.230–0.295)	0.23 (0.19–0.27)	0.60 (0.43–0.75)	1.35 (1.16–1.53)	2.18 (1.53–3.26)	697	68.1
2001–2002^a^	0.290 (0.233–0.361)	0.26 (0.19–0.35)	0.65 (0.49–0.81)	1.26 (0.86–2.17)	2.65 (1.50–3.44)	767	76
1999–2000^b^	0.234 (0.202–0.272)	0.16 (0.13–0.19)	0.31 (0.27–0.39)	0.64 (0.49–0.95)	1.06 (0.67–1.71)	697	68.1
2001–2002^b^	0.274 (0.230–0.328)	0.20 (0.18–0.24)	0.39 (0.31–0.48)	0.73 (0.58–0.96)	1.18 (0.79–1.98)	767	76

Non-Hispanic black							
1999–2000^a^	0.454 (0.352–0.586)	0.45 (0.35–0.61)	1.13 (0.75–1.46)	2.32 (1.45–5.35)	5.35 (2.32–21.1)	524	80.3
2001–2002^a^	0.507 (0.430–0.599)	0.52 (0.44–0.63)	0.98 (0.84–10.20)	2.03 (1.72–2.52)	3.86 (3.05–5.84)	776	88.7
1999–2000^b^	0.309 (0.238–0.401)	0.25 (0.20–0.32)	0.58 (0.39–0.78)	1.36 (0.89–2.79)	3.43 (1.39–5.44)	524	80.3
2001–2002^b^	0.374 (0.308–0.455)	0.32 (0.25–0.39)	0.59 (0.50–0.69)	1.29 (0.93–1.66)	2.62 (1.58–3.26)	775	88.7

< LOD indicates less than the LOD for the uncorrected urine values, which may vary for some chemicals by year. *n*-Values may differ because some information was missing for a particular subgroup.

a Volume-based concentrations (ng/mL).

b Creatinine-adjusted concentrations (μg/g creatinine).

**Table 3:** Urinary concentrations of *cis* DCCA in the general U.S. population, NHANES 1999–2002.

Category/survey years	GM (95% confidence interval)	Selected percentile (95% confidence interval)				Sample size (*n*)	Weighted detection (%)
		50th	75th	90th	95th		
Total							
1999–2000^a^	NC	< LOD	0.27 (0.22–0.34)	0.60 (0.49–0.71)	1.12 (0.77–1.68)	1,951	46.7
2001–2002^a^	NC	< LOD	0.17 (0.13–0.23)	0.51 (0.41–0.67)	0.91 (0.80–1.10)	3,048	32.4
1999–2000^b^	NC	< LOD	0.26 (0.23–0.29)	0.54 (0.44–0.70)	1.12 (0.69–1.59)	1,951	46.7
2001–2002^b^	NC	< LOD	0.23 (0.21–0.25)	0.46 (0.42–0.54)	0.90 (0.75–1.04)	3,046	32.4

Age group							
6–11 years							
1999–2000^a^	NC	< LOD	0.33 (0.21–0.55)	0.74 (0.58–1.53)	1.77 (0.68–3.15)	468	46.6
2001–2002^a^	NC	< LOD	0.11 (< LOD-0.20)	0.37 (0.28–0.61)	0.73 (0.49–0.87)	580	26.4
1999–2000^b^	NC	< LOD	0.26 (0.19–0.40)	0.63 (0.40–1.38)	1.38 (0.59–1.80)	468	46.6
2001–2002^b^	NC	< LOD	0.21 (< LOD-0.25)	0.40 (0.32–0.58)	0.70 (0.54–0.77)	580	26.4

12–19 years							
1999–2000^a^	NC	< LOD	0.30 (0.20–0.41)	0.67 (0.46–1.11)	1.44 (0.67–2.21)	667	48
2001–2002^a^	NC	< LOD	0.16 (< LOD–0.21)	0.44 (0.30–0.63)	0.73 (0.63–0.92)	831	31.7
1999–2000^b^	NC	< LOD	0.16 (0.13–0.21)	0.32 (0.25–0.45)	0.57 (0.32–1.24)	667	48
2001–2002^b^	NC	< LOD	0.13 (< LOD-0.18)	0.27 (0.21–0.33)	0.38 (0.29–0.56)	830	31.7

20–59 years							
1999–2000^a^	NC	< LOD	0.26 (0.20–0.33)	0.57 (0.43–0.69)	1.07 (0.67–1.80)	816	46.5
2001–2002^a^	NC	< LOD	0.17 (0.12–0.23)	0.51 (0.40–0.74)	0.96 (0.79–1.28)	1,128	32.6
1999–2000^b^	NC	< LOD	0.29 (0.26–0.32)	0.55 (0.44–1.06)	1.33 (0.83–1.75)	816	46.5
2001–2002^b^	NC	< LOD	0.26 (0.24–0.28)	0.58 (0.44–0.72)	1.05 (0.80–1.46)	1,128	32.6

≥ 60 years							
2001–2002^a^	NC	< LOD	0.22 (0.13–0.35)	0.64 (0.38–1.29)	1.77 (0.76–3.10)	509	36
2001–2002^a^	NC	< LOD	0.31 (0.24–0.37)	0.68 (0.42–1.23)	1.39 (0.72–2.65)	508	36

Sex							
Male							
1999–2000^a^	NC	< LOD	0.25 (0.20–0.31)	0.53 (0.42–0.60)	0.79 (0.60–1.50)	947	46.8
2001–2002^a^	NC	< LOD	0.16 (0.12–0.21)	0.49 (0.41–0.64)	1.03 (0.70–1.31)	1,429	31.6
1999–2000^b^	NC	< LOD	0.22 (0.18–0.26)	0.41 (0.35–0.57)	0.80 (0.51–1.11)	947	46.8
2001–2002^b^	NC	< LOD	0.19 (0.17–0.21)	0.41 (0.33–0.47)	0.74 (0.62–1.05)	1,429	31.6

Female							
1999–2000^a^	NC	< LOD	0.28 (0.22–0.38)	0.68 (0.49–1.08)	1.47 (0.95–2.54)	1,004	46.6
2001–2002^a^	NC	< LOD	0.19 (0.12–0.25)	0.56 (0.40–0.74)	0.90 (0.79–1.13)	1,619	33.2
1999–2000^b^	NC	< LOD	0.2 (0.27–0.34)	0.63 (0.47–1.33)	1.55 (1.00–2.03)	1,004	46.6
2001–2002^b^	NC	< LOD	0.27 (0.23–0.30)	0.52 (0.46–0.63)	0.96 (0.84–1.20)	1,617	33.2

Race/ethnicity							
Non-Hispanic white							
1999–2000^a^	NC	< LOD	0.27 (0.22–0.34)	0.63 (0.46–0.78)	1.13 (0.74–2.35)	591	46.3
2001–2002^a^	NC	< LOD	0.16 (< LOD–0.23)	0.51 (0.38–0.76)	0.96 (0.79–1.17)	1,281	29.6
1999–2000^b^	NC	< LOD	0.31 (0.28–0.40)	0.83 (0.55–1.33)	1.55 (1.06–1.99)	591	46.3
2001–2002^b^	NC	< LOD	0.28 (< LOD–0.32)	0.63 (0.50–0.71)	1.14 (0.96–1.38)	1,280	29.6

Mexican American							
1999–2000^a^	NC	< LOD	0.20 (0.11–0.24)	0.46 (0.30–0.61)	0.73 (0.47–1.32)	671	35.9
2001–2002^a^	NC	< LOD	0.14 (0.11–0.18)	0.30 (0.25–0.39)	0.51 (0.38–0.64)	767	31.3
1999–2000^b^	NC	< LOD	0.14 (0.11–0.21)	0.29 (0.23–0.35)	0.40 (0.29–0.67)	671	35.9
2001–2002^b^	NC	< LOD	0.16 (0.14–0.19)	0.28 (0.25–0.30)	0.38 (0.33–0.50)	767	31.3

Non-Hispanic black							
1999–2000^a^	0.202 (0.155–0.262)	0.16 (0.12–0.20)	0.38 (0.27–0.52)	0.82 (0.49–1.68)	1.68 (0.91–5.43)	518	61.5
2001–2002^a^	NC	< LOD	0.30 (0.24–0.37)	0.67 (0.53–0.78)	0.94 (0.77–1.37)	776	48.5
1999–2000^b^	0.138 (0.104–0.182)	0.11 (0.08–0.16)	0.23 (0.18–0.28)	0.46 (0.29–0.94)	1.02 (0.52–1.80)	518	61.5
2001–2002^b^	NC	< LOD	0.17 (0.14–0.20)	0.35 (0.26–0.48)	0.58 (0.37–1.39)	775	48.5

NC, not calculated (proportion of results below LOD was too high to provide a valid result).

< LOD indicates less than the LOD for the uncorrected urine values, which may vary for some chemicals by year. *n*-Values may differ because some information was missing for a particular subgroup.

a Volume-based concentrations (ng/mL).

b Creatinine-adjusted concentrations (μg/g creatinine).

**Table 4:** Urinary concentrations of *trans*-DCCA in the general U.S. population, NHANES 1999–2002.

Category/survey years	Selected percentile (95% confidence interval)			Sample size (*n*)	Weighted detection (%)
	75th	90th	95th		
Total					
1999–2000^a^	0.56 (0.48–0.70)	1.40 (1.17–1.77)	3.42 (2.39–5.56)	1,976	33.1
2001–2002^a^	0.43 (< LOD–0.56)	1.18 (0.93–1.64)	2.60 (1.85–3.58)	3,033	26.4
1999–2000^b^	0.70 (0.61–0.78)	1.56 (1.33–1.87)	2.65 (2.15–3.89)	1,976	33.1
2001–2002^b^	0.74 (< LOD–0.80)	1.47 (1.32–1.87)	2.62 (2.30–3.11)	3,031	26.4

Age group					
6–11 years					
1999–2000^a^	0.97 (0.70–10.66)	2.91 (1.76–4.19)	4.19 (2.97–11.7)	478	42
2001–2002^a^	0.47 (< LOD–0.76)	1.39 (1.03–1.68)	2.50 (1.55–3.54)	576	28.8
1999–2000^b^	0.90 (0.64–1.39)	1.85 (1.17–3.77)	3.67 (1.55–9.49)	478	42
2001–2002^b^	0.77 (< LOD–0.97)	1.40 (1.12–2.16)	2.55 (1.50–3.11)	576	28.8

12–19 years					
1999–2000^a^	0.71 (0.52–0.86)	2.07 (1.25–3.42)	4.28 (2.12–6.23)	675	39.2
2001–2002^a^	0.49 (< LOD–0.67)	1.20 (0.80–1.60)	2.01 (1.49–3.77)	826	31.3
1999–2000^b^	0.47 (0.38–0.55)	1.02 (0.70–1.42)	1.56 (0.96–4.02)	675	39.2
2001–2002^b^	0.47 (< LOD–0.58)	0.85 (0.71–1.07)	1.29 (1.00–1.63)	825	31.3

20–59 years					
1999–2000^a^	0.50 (0.40–0.62)	1.17 (0.91–1.68)	2.94 (1.49–5.56)	823	30.4
2001–2002^a^	< LOD	1.17 (0.85–1.85)	2.56 (1.64–4.66)	1,123	24.7
1999–2000^b^	0.74 (0.67–1.00)	1.75 (1.29–2.26)	3.13 (2.22–4.48)	823	30.4
2001–2002^b^	< LOD	1.87 (1.37–2.33)	2.89 (2.34–3.50)	1,123	24.7

≥ 60 years					
2001–2002^a^	0.46 (< LOD–0.63)	1.13 (0.76–2.44)	4.27 (1.20–6.49)	508	27.9
2001–2002^a^	0.86 (< LOD–0.97)	1.92 (1.30–2.71)	3.31 (2.03–5.33)	507	27.9

Sex					
Male					
1999–2000^a^	0.56 (0.50–0.67)	1.28 (1.11–1.63)	2.25 (1.55–5.10)	961	33.7
2001–2002^a^	0.42 (< LOD–0.50)	1.12 (0.86–1.58)	2.44 (1.69–3.70)	1,419	26.1
1999–2000^b^	0.57 (0.50–0.67)	1.23 (1.05–1.35)	2.15 (1.53–2.74)	961	33.7
2001–2002^b^	0.57 (< LOD–0.61)	1.22 (1.00–1.45)	2.33 (1.68–2.73)	1,419	26.1

Female					
1999–2000^a^	0.55 (0.41–0.82)	1.77 (1.07–3.08)	4.19 (3.08–6.81)	1,015	32.6
2001–2002^a^	0.44 (< LOD–0.63)	1.23 (0.91–1.98)	2.62 (1.90–3.58)	1,614	26.7
1999–2000^b^	0.88 (0.72–1.11)	1.87 (1.47–2.37)	3.65 (2.26–5.94)	1,015	32.6
2001–2002^b^	0.85 (< LOD–0.95)	1.75 (1.47–2.15)	2.98 (2.55–3.20)	1,612	26.7

Race/ethnicity					
Non-Hispanic white					
1999–2000^a^	0.56 (0.46–0.73)	1.41 (1.14–2.14)	3.89 (2.14–6.43)	595	32.7
2001–2002^a^	0.41 (< LOD–0.58)	1.18 (0.85–1.85)	2.64 (1.85–4.27)	1,278	25.5
1999–2000^b^	1.00 (0.76–1.12)	2.15 (1.65–2.74)	4.48 (2.64–6.29)	595	32.7
2001–2002^b^	0.88 (< LOD–0.99)	2.14 (1.57–2.33)	3.16 (2.63–3.50)	1,277	25.5

Mexican American					
1999–2000^a^	0.47 (0.41–0.53)	1.23 (0.83–1.60)	1.87 (1.49–3.35)	691	30.2
2001–2002^a^	0.41 (< LOD–0.51)	0.86 (0.68–1.14)	1.57 (1.08–2.01)	767	25.8
1999–2000^b^	0.52 (0.44–0.64)	1.01 (0.80–1.17)	1.47 (1.17–1.69)	691	30.2
2001–2002^b^	0.59 (< LOD–0.67)	1.04 (0.90–1.17)	1.40 (1.17–1.87)	767	25.8

Non-Hispanic black					
1999–2000^a^	0.78 (0.49–1.13)	1.84 (1.08–4.69)	4.69 (1.41–14.5)	518	41
2001–2002^a^	0.59 (0.49–0.74)	1.26 (1.05–1.70)	2.25 (1.54–3.32)	764	36.7
1999–2000^b^	0.50 (0.41–0.68)	1.08 (0.76–2.15)	2.15 (1.16–3.96)	518	41

< LOD indicates less than the limit of detection for the uncorrected urine values, which may vary for some chemicals by year. The 50th percentile measures were all < LOD and are not shown here. GMs were not calculated (proportion of results below limit of detection was too high to provide any valid results). *n*-Values may differ if some information was missing for a particular subgroup.

a Volume-based concentrations (ng/mL).

b Creatinine-adjusted concentrations (μg/g creatinine).
